# Sistemas nacionais de saúde, legislação e seus determinantes sociais: um estudo comparativo entre Brasil, Espanha, Portugal e Itália

**DOI:** 10.1590/0102-311XPT169423

**Published:** 2024-07-29

**Authors:** Simone de Pinho Barbosa, José Ramon Martinez-Riera, Tereza Maria Mendes Diniz de Andrade Barroso, Ivan Hernadez-Caravaca, Aliete Cunha Oliveira, César Iván Avilés González, Manuela Racis, Marcos Alex Mendes da Silva, Daniela Lourenço Pinto, Álvaro Luiz Fonseca Campos, Lawrence Monteiro Pio, Francisco Carlos Félix Lana

**Affiliations:** 1 Universidade Federal de Juiz de Fora, Governador Valadares, Brasil.; 2 Universidad de Alicante, Alicante, España.; 3 Escola Superior de Enfermagem de Coimbra, Coimbra, Portugal.; 4 Università Degli Studi di Cagliari, Cagliari, Italia.; 5 Azienda Socio-sanitaria del Medio Campidano, Sanluri, Italia.; 6 Universidade Federal Fluminense, Nova Friburgo, Brasil.; 7 Universidade Federal de Minas Gerais, Belo Horizonte, Brasil.

**Keywords:** Sistemas Nacionais de Saúde, Determinantes Sociais da Saúde, Modelos de Assistência à Saúde, Atenção Primária à Saúde, Legislação, National Health Systems, Social Determinants of Health, Healthcare Models, Primary Health Care, Legislation, Sistemas Nacionales de Salud, Determinantes Sociales de la Salud, Modelos de Atención de Salud, Atención Primaria de Salud, Legislación

## Abstract

Trata-se de uma pesquisa documental, exploratória, descritiva, partindo de um estudo multicêntrico e internacional entre Brasil, Espanha, Itália e Portugal sobre sistemas nacionais de saúde com modelo de atenção baseado na atenção primária à saúde e financiado pelo Conselho Nacional de Desenvolvimento Científico e Tecnológico (CNPq) do Brasil. Tem como objetivo identificar as legislações de base da saúde, o direito à saúde e os princípios doutrinários e organizativos de cada país selecionado com ênfase no impacto dos determinantes sociais de saúde sobre os sistemas nacionais de saúde. Os resultados revelaram países com legislações e princípios doutrinários semelhantes, com direito à saúde constitucional, ancorados na atenção primária à saúde, e com modelo assistencial de acesso do tipo saúde da família. Os desafios encontrados foram a baixa natalidade e elevada expectativa de vida ao nascer em países europeus e critérios para acesso a medicamentos e financiamento assistencial. Com base nos nossos achados, os países que tiveram maior investimento em base estrutural, perpassando por assegurar condições socioeconômicas e sanitárias mais dignas, sólidas e vigilantes, garantiram importante diferenciação na capacidade de resposta e sustentabilidade do sistema nacional de saúde e no impacto direto na qualidade de vida das pessoas.

## Abstract (en)

This is a documentary, exploratory, descriptive study, which is part of a multicenter international study assessing the national health systems with a care model based on primary health care of Brazil, Spain, Italy, and Portugal, funded by the Brazilian National Research Council (CNPq, acronym in Portuguese). It aims to identify the basic health legislation, the right to health, and the doctrinal and organizational principles of each country with a focus on the impact of social determinants of health on the national health systems. The results showed these countries have similar legislation and doctrinal principles, with a constitutional right to health, based on primary health care, and with a care model of the family health type. The challenges identified were low birth rate and high life expectancy at birth in European countries and criteria for access to medication and care financing. Based on our findings, the countries with higher investment in a structural basis, ensuring more dignified, solid, and vigilant socioeconomic and sanitary conditions, provide an important differentiation in responsiveness and sustainability of the national health system and direct impact on the quality of life.

## Resumen

Se trata de una investigación documental, exploratoria, descriptiva, parte de un estudio multicéntrico, internacional entre Brasil, España, Italia y Portugal sobre los Sistemas Nacionales de Salud con un modelo de atención basado en la atención primaria de salud y financiado por el Consejo Nacional de Desarrollo Científico y Tecnológico (CNPq) de Brasil. Tiene como objetivo identificar la legislación de base de la salud, el derecho a la salud y los principios doctrinales y organizativos de cada país seleccionado con énfasis en el impacto de los determinantes sociales de la salud sobre los sistemas nacionales de salud. Los resultados revelaron países con legislaciones y principios doctrinales similares, con derecho a salud constitucional, anclados en la atención primaria de salud y con un modelo asistencial de acceso del tipo salud de la familia. Los desafíos encontrados fueron la baja tasa de natalidad y la alta esperanza de vida al nacer en países europeos y criterios para el acceso a medicamentos y financiación asistencial. Con base en nuestros hallazgos, los países que tuvieron mayor inversión en base estructural, asegurando condiciones socioeconómicas y sanitarias más dignas, sólidas y vigilantes, garantizan una diferenciación importante en la capacidad de respuesta y sostenibilidad del sistema nacional de salud y en el impacto directo en la calidad de vida de las personas.

## Introdução

As experiências internacionais se traduzem em uma troca de contribuições, informações, vivências e conhecimentos capazes de ampliar a cooperação e a inovação tecnológica. Os sistemas nacionais de saúde (SNS) vem adotando a atenção primária à saúde (APS) para organizar, acompanhar e ordenar os recursos, respondendo de maneira apropriada às necessidades de sua população, numa proposta cada vez mais próxima dos territórios e suas singularidades ^1^.

Dessa forma, para termos resultados promotores de qualidade de saúde e vida, é essencial que haja uma ação comprometida por governantes acerca das questões econômicas, sociais, culturais, étnicas, comportamentais e de saúde das sociedades ^2^. Compreender a importância da inovação de tecnologias em saúde tem sido uma abertura bem sucedida como forma de pensar uma saúde pública mais qualificada e abrangente, com oferta de ações e de cuidado cada vez mais complacente, promotora de autonomia dos usuários e com uso racional e eficiente dos recursos públicos ^3^.

Cabe destacar que o comportamento humano depende do contexto de cada pessoa, devendo ser aplicada a promoção da saúde a todos os contextos e realizada de forma singular de acordo com as especificidades de cada local e comunidade. É necessário criar condições que possibilitem às pessoas conquistar saúde por seu favorecimento e pelo reforço positivo dado aos comportamentos saudáveis, levando em consideração as relações causais recíprocas que envolvem fatores comportamentais, sociais, econômicos e ambientais, fortemente inter-relacionados e que atuam na criação de contextos que podem permitir, facilitar, promover, dificultar ou até mesmo impedir o acesso à saúde em tempo ideal ^2^ ^,^ ^4^.

Nessa perspectiva, os determinantes sociais de saúde (DSS) estão relacionados aos fatores sociais, econômicos, culturais, étnicos/raciais, psicológicos, comportamentais, ambientais, biológicos, estilos de vida, demográficos e relacionados com o sistema de saúde e a prestação de cuidados de saúde ^2,5^. Essas causas influenciam diretamente na ocorrência de problemas de saúde e seus fatores de risco e contribuem para o estado de saúde das populações. Países com combinações sociais frágeis são os que menos investem em capital humano e no fortalecimento de arranjos organizativos acerca das políticas públicas, da cidadania e sobretudo das coletividades fundamentais para o desenvolvimento humano: a promoção e proteção da saúde nos âmbitos individual e coletivo. É imperativo destacar que não são as sociedades mais ricas que produzem melhores resultados nas condições de saúde e vida, mas as mais igualitárias e com alta coesão social ^2^ ^,^ ^5^.

Para tanto, a conformação dos SNS deve perpassar pelos modelos assistenciais pautados na promoção da saúde, considerando o perfil das nações, de suas populações e suas necessidades e também dos projetos de governo que regem essas escolhas, o que agencia a inovação tecnológica e o avanço na qualidade da oferta do cuidado em saúde, na capacidade de respostas às novas demandas e na sustentabilidade operacional dos SNS. Esses modelos assistenciais geram significativos efeitos nos *modus operandi* dos serviços de saúde e na resposta à peculiaridade da vida social, que demanda o desenvolvimento de novas ofertas para os serviços, de novas ações, programas e habilidades para o avanço da produção em saúde e sobretudo da capacidade dos profissionais no agir, com intuito de lidar cada vez mais com a complexidade das novas demandas sociossanitárias do trabalho em saúde ^3^.

Ao estar mais próxima da população, a APS aborda problemas singulares das comunidades com tecnologias voltadas à promoção e proteção da saúde, à prevenção dos agravos, ao tratamento e à reabilitação apropriados às demandas. Esse nível de atenção estabelece e racionaliza o uso de todos os recursos, tanto básicos como especializados e internações, sobretudo pela aptidão de compreender intimamente às necessidades comunitárias com melhor impacto em saúde ^4^ ^,^ ^6^ ^,^ ^7^.

O aumento na aquisição de recursos humanos nos sistemas de saúde como opção isolada não é razoável se não for associada à modificação do produzir e do agir em saúde, além do fato de que implantar novas equipes de saúde da família não garante a mudança das práticas de saúde nem a integralidade da assistência, pois o modelo assistencial não certifica o sucesso por si só, mas sim a possibilidade de melhorias na qualidade das ações sanitárias desde que as diversas arestas comecem a ser aparadas ^8^ ^,^ ^9^.

Considerando esse desafio, foi questionado se o investimento em tecnologia de saúde do tipo processo, também conhecida como tecnologias sociais ou alternativas, favorecem a APS e a sustentabilidade dos SNS com cobertura universal.

Um modelo assistencial de base mais forte que se aproxime do território e a adoção de novas ações que componham um conjunto de habilidades do fazer saúde que promova novos avanços, se fazem cada vez mais necessários ao progresso dos SNS. Critérios normativos que assegurem esse tipo de abordagem junto à qualificação dos recursos humanos com desenvolvimento de competências comunitárias, capazes de elaborar novos formatos “intertecnológicos” do atuar em saúde, com máxima interação com as pessoas e o território, são primordiais. Para que isso ocorra, é necessário compreender as estruturas que alicerçam uma nação, com projetos de proteção social mais robustos que promovam de fato as transformações necessárias ao fortalecimento das condições de vida de uma sociedade.

Dessa maneira, entendendo a importância de compreender o direito à saúde, as doutrinas, as diretrizes e os princípios que regem os SNS e os DSS, este estudo teve como objetivo comparar as normativas gerais regentes que conformam esses sistemas e indicadores de base socioeconômica e de saúde, buscando elucidar fatores determinantes do avanço da APS e, consequentemente, dos SNS.

## Metodologia

Estudo multicêntrico e comparativo, entre Brasil, Espanha, Itália e Portugal, sobre sistemas nacionais de saúde e inovação tecnológica para a atenção primária à saúde. A pesquisa foi realizada por meio de métodos mistos e executada em três etapas: a primeira foi de pesquisa documental; a segunda, de observação direta participante em 21 serviços de APS; e a terceira, de entrevistas com 75 profissionais de saúde atuantes nesses serviços. A aprovação pelo comitê de ética da Universidade Federal de Juiz de Fora se deu sob o parecer consubstanciado nº 5.399.351, no Brasil, sendo financiada pelo Conselho Nacional de Desenvolvimento Científico e Tecnológico (CNPq) por meio do Programa de Pós-Doutorado no Exterior (PDE).

Este artigo se refere à pesquisa documental, de caráter exploratório e descritivo, realizada no período de março de 2022 a março de 2023, com seleção de legislações regulatórias dos sistemas nacionais de saúde do Brasil, da Espanha, de Portugal e da Itália, em conformidade com o interesse da hipótese e do objetivo do estudo. Foi utilizado roteiro para orientar a extração dos dados documentais, tendo como objeto a investigação do direito à saúde, dos princípios doutrinários, dos organizativos e das diretrizes dos SNS e do modelo de atenção vigente adotado na APS. A análise foi feita a partir de consensos e diferenças encontrados e posteriormente contrastadas com as literaturas especializadas nos temas. As fontes foram extraídas das homepages oficiais do Ministério da Saúde dos países selecionados e do *Diário Oficial da União* (DOU) do Brasil, do *Boletín Oficial del Estado* (BOE) da Espanha, do *Diário da República* (DR) de Portugal e da *Gazzetta Ufficiale* (GU) da Itália.

Quanto às fontes secundárias, foram utilizadas e consultadas um total de 10 diferentes tabuladores de dados de domínio público dos países investigados, com seleção de indicadores da situação socioeconômica e sanitária relacionados aos determinantes sociais de saúde. Foram elaboradas três seções para a discussão das legislações estudadas e suas proposições teórico-administrativas: o direito à saúde; os sistemas nacionais de saúde - aspectos históricos, características e rede de serviços; e os determinantes sociais de saúde - análise imperativa na qualidade dos sistemas nacionais de saúde. Assim, podemos refletir sobre o avanço dos serviços de saúde e sobre a força dos DSS para a capacidade de produção e de respostas dos SNS.

## Resultados e discussão

O conjunto documental selecionado é composto de 36 documentos e 11 diferentes bancos de dados secundários: Programa das Nações Unidas para o Desenvolvimento (PNUD); Organização para a Cooperação e Desenvolvimento Econômico (OCDE); Banco Mundial (The World Bank Data); Instituto Brasileiro de Geografia e Estatística (IBGE); Organização Mundial da Saúde (SCORE country profile); The Statistic Portal for Market Data (STATISTA, portal estatístico para dados de mercado); Istituto Nazionale di Statistica (ISTAT, Instituto Nacional de Estatística, da Itália); The World Factbook, da Central Intelligence Agency (CIA, Agência Central de Inteligência, dos Estados Unidos); Facts and Policies of the Italian National Health Services (Fatos e Políticas dos Serviços Nacionais de Saúde Italianos), do Ministério da Saúde Italiano; The European Observatory on Health and Policies (Observatório Europeu dos Sistemas e Políticas de Saúde); e o banco de dados Atlas Mundial da plataforma Knoema. 

Os documentos foram tratados, codificados e catalogados de acordo com o país, nome, ano de publicação e seus conteúdos, conforme o Quadro 1.

**Quadro 1:** Catalogação e síntese dos documentos investigados. Brasil, Espanha, Itália e Portugal, 2023.

CÓDIGO	PAÍS	DOCUMENTOS	ANO	SÍNTESE DOS DOCUMENTOS
D01	Espanha	*Constituição Espanhola*	1978	Institui o direito à saúde sobre equidade, integralidade, universalidade. Art. 43
D02	Espanha	*Lei Geral da Saúde nº 14*	1986	Tem por objeto a regulamentação geral de todas as ações que permitam a efetivação do direito à proteção da saúde reconhecido no art. 43 e concordante da Constituição
D03	Espanha	*Decreto-Lei nº 1.030*	2006	Estabelece o conteúdo da carteira de serviços comuns de saúde pública, cuidados primários, cuidados especializados, cuidados de urgência, farmacêuticos, ortopédicos, produtos dietéticos e serviços de transporte de saúde e a garantia da equidade e acessibilidade aos cuidados de saúde
D04	Espanha	*Carteira de Serviços de Atenção Primaria*	2010	Dispõe sobre o desenvolvimento, a organização, o uso e conteúdo de ações da APS
D05	Espanha	*Decreto-Lei nº 16*	2012	Trata de medidas urgentes para garantir a sustentabilidade do SNS e melhorar a qualidade e a segurança dos seus serviços
D06	Espanha	*Decreto-Lei nº 7*	2018	Dispõe sobre o acesso universal ao SNS
D07	Espanha	*Marco Estratégico para a Atenção Primaria a Saúde*	2019	Institui a urgência em promover a atualização dos cuidados de saúde primários e comunitários do SNS
D08	Portugal	*Decreto-Lei n.º 413*	1971	Institui os centros de saúde de primeira geração
D09	Portugal	*Constituição Portuguesa* (art. 64)	1976	Institui o direito à saúde sobre equidade, integralidade, universalidade
D10	Portugal	*Lei nº 56*	1979	Criação do SNS, concretizando o direito à proteção da saúde, a prestação de cuidados globais de saúde e o acesso a todos os cidadãos, independentemente da sua condição econômica e social
D11	Portugal	*Decreto-Lei nº 254*	1982	Criação das ARS, que sucedem às administrações distritais dos serviços de saúde
D12	Portugal	*Decreto-Lei nº 74*	1984	Criação da Direção-Geral dos Cuidados de Saúde Primários, com funções de orientação técnico-normativa, pelos órgãos e serviços regionais, distritais e locais que intervêm na área dos cuidados de saúde primários. O clínico geral adquire o estatuto de médico de família
D13	Portugal	*Lei de Bases da Saúde* (n.º 48)	1990	A proteção da saúde passa a ser mais que um direito: é assumida como uma responsabilidade conjunta dos cidadãos, da sociedade e do Estado, em liberdade de procura e de prestação de cuidados
D14	Portugal	*Decreto-Lei nº 11*	1993	Trata da hierarquização do Serviço Nacional de Saúde sob a tutela do Ministério da Saúde, com o objetivo de efetivar, por parte do Estado, a responsabilidade de proteção à saúde individual e coletiva
D15	Portugal	*Decreto-Lei nº 157*	1999	Estabelece o regime de criação, organização e funcionamento dos centros de saúde
D16	Portugal	*Decreto-Lei nº 60*	2003	Criação da Rede de Cuidados de Saúde Primários
D17	Portugal	*Decreto-Lei nº 88*	2005	Dispõe sobre a gestão de pessoal dirigente a exercer funções ao abrigo do *Decreto-Lei nº 60/2003*. Revoga o *Decreto-Lei nº 60*, de 1 de abril de 2003, que cria a rede de cuidados de saúde primários, e repristina o *Decreto-Lei nº157*, de 10 de maio de 1999, que estabelece o regime de criação, organização e funcionamento dos centros de saúde
D18	Portugal	*Lei nº 95*	2019	Aprova a Lei de Bases da Saúde e revoga a *Lei nº 48*, de 24 de agosto de 1990, e o *Decreto-Lei nº 185*, de 20 de agosto de 2002
D19	Itália	*Constituição da República Italiana* (art. 32)	1947	A república tutela a saúde como direito fundamental do indivíduo e interesse da coletividade e garante tratamentos gratuitos aos indigentes. Ninguém pode ser obrigado a um determinado tratamento sanitário, salvo disposição de lei. A lei não pode, em hipótese alguma, violar os limites impostos pelo respeito à pessoa humana
D20	Itália	*Lei nº 405*	1975	Criação dos Centros de Aconselhamento Familiar, *Consultori Familliare*, o serviço de assistência à família e à maternidade
D21	Itália	*Lei nº 833*	1978	Criação do Sistema Nacional de Saúde
D22	Itália	*Decreto-Lei nº 502*	1992	Definição dos níveis essenciais de assistência - LEA
D23	Itália	*Decreto-Lei nº 33*	2001	Definição dos níveis essenciais de assistência - LEA
D24	Itália	*Decreto-Lei nº 65*	2017	Definição dos níveis essenciais de assistência - LEA. Cuidados Primários em Saúde
D25	Itália	*Plano Nacional de Prevenção*	2020	A emergência de saúde devido à pandemia de COVID-19 mostrou que as intervenções de saúde pública são fundamentais para o desenvolvimento econômico e social de um país e da saúde de todos depende da saúde de cada um. O Plano Nacional de Prevenção (PNP) representa o quadro comum dos objetivos de muitas das áreas relevantes para a saúde pública
D26	Itália	*Decreto-Lei nº 77*	2022	Dispõe sobre assistência territorial, padrões de qualidade, estruturais, tecnológicos e quantidades, ao monitoramento, as regiões de saúde e a sustentabilidade financeira
D27	Itália	*Disposição Geral nº 33*	2022	Sobre o planejamento da assistência territorial de acordo com o *Decreto-Lei nº 77/2022*
D28	Brasil	*Constituição da República Federativa do Brasil*	1988	Capitulo da Saúde: institui o direito a saúde sobre equidade, integralidade, universalidade, participação popular, regionalização, descentralização com ênfase na municipalização, resolutividade, hierarquização e estabelece o conceito ampliado de saúde
D29	Brasil	*Lei nº 8.080*	1990	Saúde é um direito de todos e dever do estado com uso do setor privado de forma complementar. Define a integralidade das ações como sendo a promoção, proteção e reabilitação da saúde e prevenção de doenças e diz que todos somos iguais acerca do direito à saúde, levando em consideração as necessidades de cada um postulado pelo princípio doutrinário da equidade. Enfatiza a gestão no âmbito municipal com descentralização do estado e da união
D30	Brasil	*Lei nº 8.142*	1990	Dispõe sobre a participação da comunidade na gestão do SUS e sobre as transferências intergovernamentais de recursos financeiros na área da saúde e dá outras providências
D31	Brasil	*Decreto nº 7.508*	2011	Regulamenta a *Lei nº 8.080* e dispõe sobre a organização do SUS, o planejamento da saúde, a assistência à saúde e a articulação interfederativa, e dá outras providências
D32	Brasil	*Lei Complementar nº 141*	2012	Trata sobre os valores mínimos a serem aplicados anualmente pela União, Estados, Distrito Federal e Municípios em ações e serviços públicos de saúde; estabelece os critérios de transferências de recursos para a saúde e as normas de fiscalização, avaliação e controle das despesas com saúde nas três esferas de governo; Regulamenta o 3^o^ parágrafo do art. 198 da *Constituição Federal* do Brasil
D33	Brasil	*Portaria nº 648*	2006	Política Nacional de Atenção Básica
D34	Brasil	*Portaria nº 2.488*	2011	Política Nacional de Atenção Básica
D35	Brasil	*Portaria nº 2.436*	2017	Política Nacional de Atenção Básica
D36	Brasil	*Carteira de Ações e Serviços da Atenção Primária em Saúde*	2019	Documento que visa nortear as ações de saúde na APS brasileira com forte reconhecimento da clínica multiprofissional. É um documento orientador para todos os serviços de APS no Brasil

APS: atenção primária à saúde; ARS: Administrações Regionais de Saúde; LEA: níveis essenciais de atenção (*Livelli Essenziali di Assistenza*); SNS: Sistema Nacional de Saúde.

Fonte: elaboração própria.

Esses documentos permitiram apontamentos e comparações quanto à garantia da saúde constitucional, a lei geral de base com princípios doutrinários e organizativos dos SNS, decretos de lei e portarias de criação de serviços de atenção primária com diretrizes referentes ao funcionamento da rede de atenção à saúde.

### O direito à saúde

O direito à saúde está diretamente ligado à liberdade, à igualdade, à participação popular das comunidades e à capacidade do Estado em garantir ao indivíduo um completo bem-estar físico, mental e social ^10^. Para confirmar a influência determinante do meio sobre a saúde, com ressalva à utópica proposição de um “completo bem estar”, cabe aqui sustentar a imagem objetiva do referido conceito, conforme mencionado por Dejours ^11^, às várias críticas reflexivas do estado de completo bem-estar, incluindo a sua não existência, contudo alegando a importância de que a saúde deve ser entendida como a busca constante de tal estado, a preservação desse desígnio movimenta as nações para entregas e alcances cada vez mais abrangentes e equitativos à sociedade ^11^. Igualmente, para garantir saúde a todos, é necessário não impedir as pessoas de procurarem seu bem-estar ou induzi-las ao adoecimento e, por isso, precisamos de instrumentos normativos regulatórios voltados à proteção e à promoção da saúde, referentes à vacinação, à notificação, ao tratamento, ao isolamento de certas doenças, ao descarte de alimentos deteriorados e ao controle do meio ambiente, da qualidade de água, ar e solo e das condições de trabalho ^11^.

Os países apresentam, em seus documentos constitucionais, a garantia do direito à saúde e SNS universal, igualitário, equânime e com participação popular. Na obtenção do direito à saúde, a Itália se coloca como precursora e visionária desde 1947, apresentando, no art. 32 de sua *Constituição Federal* ^12^, o seguinte: “*A República protege a saúde como direito fundamental do indivíduo e no interesse da coletividade, e garante atendimento gratuito aos pobres. Ninguém pode ser obrigado a submeter-se a determinado tratamento médico, exceto por lei. A lei não pode, em hipótese alguma, violar os limites impostos pelo respeito à pessoa humana*”. Trinta anos depois, Portugal e Espanha, em 1976 e 1978 respectivamente, apresentam a garantia de tal direito às suas sociedades, e 41 anos após a Constituição italiana, em 1988, o Brasil no art. 196 da *Constituição Federal* dispõe de tal garantia: “*a saúde é direito de todos e dever do Estado, garantido mediante políticas sociais e econômicas que visem à redução do risco de doença e de outros agravos e ao acesso universal e igualitário às ações e serviços para a promoção, proteção e recuperação*” ^13^.

É importante esclarecer que direito, em vários idiomas, significa ordens de conduta humana ^10^ e, por isso, é essencial a participação social e a regionalização da produção da saúde, já que a aproximação territorial agrega capacidade em delimitar a igualdade e a liberdade de cada comunidade, que juntas se conformam na universalidade do direito e na extensão e multidimensionalidade do campo da saúde ^10^ ^,^ ^14^ ^,^ ^15^.

Quanto à sustentabilidade dos SNS, os achados apontam para países com princípios semelhantes, como igualdade, equidade, atenção integral, hierarquização, participação popular, regionalização e descentralização gestora; contudo, cabe um destaque para a Espanha, que apresenta como diretrizes a eficácia, a celeridade, a economia e a flexibilidade atreladas ao funcionamento do sistema nacional de saúde. Na Itália, há o *Programma Nazionale Equità della Salute* (Programa Nacional de Equidade em Saúde), um programa que prevê o fortalecimento dos serviços de saúde a partir de um acesso mais equitativo, estando presente em sete das 20 regiões italianas: Basílica, Calabria, Campania, Molise, Puglia, Sardenha e Sicília ^14^. No Brasil, a equidade é postulada para todos os estados e municípios pela *Lei nº 8.080*, de 19 de setembro de 1990, que regulamenta o SNS ^13^ ^,^ ^15^.

Outro ponto importante identificado foi o componente financiamento, que aborda os recursos que sustentam o sistema de saúde; nesses caso, a fonte principal é tributária com gratuidade regulada e taxas de coparticipação, para Itália ^12^ e Portugal ^16^, e, para Brasil ^13^ e Espanha ^17^, com gratuidade plena para todos os níveis assistenciais.

Nesse sentido, o direito à saúde não se reduz apenas ao acesso a serviços de saúde, embora eles tenham grande valor como direito fundamental. Ele vai muito além, pois implica na entrega efetiva e ampla do maior grau de qualidade de saúde e vida ao qual as pessoas possam ter acesso, o que direciona uma interface com outros direitos básicos, como educação, saneamento básico, lazer, trabalho, moradia, alimentação e segurança pública ^12^ ^,^ ^18^ ^,^ ^19^, como dito no art. 25 da Declaração Universal dos Direitos Humanos: “*Todo ser humano tem direito a um padrão de vida capaz de assegurar a si e à sua família, saúde, bem-estar, inclusive alimentação, vestuário, habitação, cuidados médicos e os serviços sociais indispensáveis*” ^18^.

Considerando a amplitude do campo da saúde e suas interfaces, está posto como disposição inicial temática analítica para os SNS, junto a seus componentes, funções e tecnologias, a garantia do direito à saúde, das estruturas que dão proteção à sociedade e ao alcance de bens e de serviços essenciais à qualidade de saúde e sobretudo à dignidade da vida humana.

### Os sistemas nacionais de saúde - aspectos históricos, características e rede de serviços

Para entendermos a organização dos sistemas de saúde, é preciso atentar para acesso, cobertura, financiamento, ciência e tecnologia incorporadas, rede de serviços assistenciais e recursos humanos disponíveis ^3^ ^,^ ^8^.

A OMS define três diferentes níveis de atenção à saúde: o primário, o secundário e o terciário. Essas categorias seguem uma ordem crescente para garantir que cada paciente seja atendido no nível correspondente, estabelecendo-se, assim, uma prioridade no acolhimento. Os achados documentais mostraram uma divisão dos níveis das assistências de saúde em redes diferentes nos países investigados. No Brasil, os níveis são divididos em primário, secundário e terciário e, nos países europeus, apenas em primário, ou de base, e especializado ou secundário ^20^ ^,^ ^21^, conforme Quadro 2. Cabe ressaltar que a nomenclatura para designar o nível primário de atenção à saúde varia entre os países: no Brasil e Espanha, é atenção primária à saúde; em Portugal, nível de cuidados de saúde primários; e assistência de base, como é conhecido na Itália. Contudo, todos possuem como modelo assistencial de primeiro contato no SNS as equipes de saúde da família, como são conhecidas no Brasil, Espanha e Portugal, e os consultórios familiares ou médicos de família ou médicos de base, na Itália; entretanto, todos adotam a divisão de clientela adscrita por equipe para organizar os atendimentos ^15^ ^,^ ^20^ ^,^ ^22^ ^,^ ^23^.

**Quadro 2:** Características dos sistemas de saúde. Brasil, Espanha, Itália e Portugal, 2023.

CARACTERÍSTICAS	BRASIL	ESPANHA	ITÁLIA	PORTUGAL
Tipo de sistema	Seguridade social	Seguridade social	Seguridade social	Seguridade social
Data de criação	1990	1986	1978	1979
Direito a saúde	Constitucional	Constitucional	Constitucional	Constitucional
Financiamento	Tributário	Tributário	Tributário com *ticket* sanitário	Tributário com taxa moderadora
Acesso	Universal/APS	Universal/APS	Universal/APS	Universal/APS
Condição de acesso	Direito à cidadania	Direito à cidadania	Direito à cidadania	Direito à cidadania
Organização	Pública	Pública	Pública	Pública
Prestação de serviços	Pública e privada	Pública	Pública e privada	Pública e privada

APS: atenção primária à saúde.

Fonte: elaboração própria.

Os países estudados apresentam forte conexão no empenho funcional das redes de atenção e serviços à saúde, buscando garantir a integralidade por meio da continuidade do cuidado. Todos têm garantia de continuidade dos cuidados de forma integral de acordo com a necessidade dos casos em diferentes densidades tecnológicas ^15^ ^,^ ^16^ ^,^ ^17^ ^,^ ^20^ ^,^ ^23^ ^,^ ^24^ ^,^ ^25^.

Quanto ao financiamento do SNS, todos são de arrecadação tributária, contudo há critérios de isenção de pagamentos conforme estabelecidos pelas normativas regulamentares, sendo a atenção de primeiro contato isenta de quaisquer pagamentos na APS dos quatro países. Brasil e Espanha têm gratuidade plena, já Itália e Portugal, gratuidade regulada, com adoção de taxas de coparticipação para pagamentos da assistência especializada, chamada de Ticket Sanitário na Itália e de Taxa Moderadora em Portugal ^26^ ^,^ ^27^. O Quadro 2 apresenta a organização, o financiamento, a condição de acesso, a oferta de serviços e o tipo de sistemas de saúde dos países relacionados.

Na Espanha, adotou-se uma reforma organizativa e desmantelou o antigo Instituto de Proteção Social (INP, Instituto de Protección Social) herdado da ditadura franquista sendo instituído: o Instituto Nacional da Saúde (INSALUD, Instituto Nacional de la Salud) para cuidados de saúde; o Instituto Nacional do Seguro Social (INSS, Instituto Nacional de la Seguridad Social) para pensões e outras transferências temporárias de dinheiro; Instituto dos Idosos e dos Serviços Sociais (IMSERSO, Instituto de Mayores e Servicios Sociales) para os serviços sociais; e Instituto Nacional de Emergência Médica (INEM) por subsídios e políticas de desemprego ^28^. A partir da década de 1980, a Espanha passou a experimentar reformas modernizadoras e universalizantes na proteção social, sobretudo a partir da segunda metade da década, com a entrada na Comunidade Europeia. Em 1986, sob a segunda legislatura socialista após a democratização, foi criado o serviço nacional de saúde ^29^.

Em Portugal, a Constituição foi estabelecida em 1976 e, por meio do art. 64, designou a saúde como direito público e seu acesso pelo Estado, independentemente de sua condição econômica e com garantia de equidade, mas a criação do SNS só ocorreu em 1979, com a *Lei de Bases de Saúde nº 59/1979* ^25^.

O Brasil figura como um importante centro político-econômico, sendo uma potência em extensão territorial, recursos naturais e diversidade cultural ^30^ ^,^ ^31^. Apesar disso, o país sofre com as mazelas clássicas dos países em desenvolvimento, como a desigualdade social, a violência urbana e a má gestão dos recursos públicos ^32^. As questões de saúde pública eram tratadas sem que houvesse uma estratégia do governo central no atendimento das demandas e a atuação do Estado restringia-se a situações emergenciais, tais como nas epidemias de centros urbanos ^31^. Em 1988, com a *Constituição Federal*, houve no cenário brasileiro a garantia da saúde e de um SNS público, universal, integral, equânime e gratuito ^30^ ^,^ ^31^.

No contexto do mundo ocidental, poucos são os países que se equiparam à importância histórica atribuída à Itália ^32^. O país figura com marcante participação em diversas frentes socioculturais, econômicas ou políticas, ter acesso gratuito à educação e ao SNS foram algumas das primeiras reformas implantadas, tendo a responsabilidade no campo da saúde compartilhada pelo Estado e suas 20 regiões administrativas ^32^ ^,^ ^33^ e a saúde como um direito fundamental aos indivíduos e ao interesse coletivo da nação, garantido pelo art. 32 da Constituição italiana em 1947 ^12^ ^,^ ^32^ ^,^ ^33^.

O SNS italiano, chamado de *Servizio Sanitario Nazionale* (SSN), foi criado em 1978 para substituir o sistema vigente que se baseava em fundos de seguro social de saúde ^31^ ^,^ ^33^. Inspirado no sistema de saúde britânico, com cobertura universal, é financiado pelos impostos nacionais e regionais, pagos por todos os cidadãos, complementados por copagamentos, principalmente a produtos farmacêuticos, exames e atendimento ambulatorial ^12^ ^,^ ^32^ ^,^ ^33^. O Estado tem competência exclusiva na fixação dos serviços ou níveis essenciais de atenção (*Livelli Essenziali di Assistenza* - LEA), enquanto as regiões supervisionam a organização e a execução de serviços primários, secundários e terciários, bem como serviços preventivos e de promoção da saúde ^33^ ^,^ ^34^. Definem seus próprios planos regionais de saúde, coordenam as estratégias das autoridades regionais de saúde, definem a alocação de recursos e o orçamento dentro de seus sistemas e monitoram a qualidade, adequação e eficiência dos serviços prestados ^33^ ^,^ ^34^.

### Determinantes sociais de saúde - análise imperativa para o avanço e qualidade dos sistemas nacionais de saúde

Ao tratarmos de SNS, é necessária uma leitura profunda sobre os elementos que determinam o feitio de uma nação e de sua população, como perfil demográfico, acesso a bens e serviços, educação, saúde, saneamento básico, distribuição de renda, força de trabalho, o nível de desigualdade social e de pobreza e políticas públicas de fortalecimento da proteção social.

Os DSS influenciam o padrão de vida e trabalho das nações, estão diretamente ligados a discussões e reflexões sobre o tema relacionado a SNS, suas variações e inovações tecnológicas, são compostos por um conjunto de fatores sociais, econômicos, ambientais, de estilo de vida, condições de saúde e têm implicações diretas nas condições de vida das pessoas de uma nação ^2^ ^,^ ^5^ ^,^ ^7^. O debate na Organização das Nações Unidas (ONU) sobre a Agenda de Desenvolvimento 2030 transcende o interesse exclusivamente global, pelos impactos que os acordos internacionais firmados no âmbito das Nações Unidas têm sobre as políticas nacionais de desenvolvimento que, por sua vez, terminam por interferir significativamente na qualidade de vida e na saúde das populações de todos os países do mundo ^35^. A Conferência das Nações Unidas de 2011 sobre o Desenvolvimento Sustentável que aconteceu no Brasil, na cidade do Rio de Janeiro, apontou para o compromisso internacional denominado “O Futuro que Queremos” (*The Future we Want*), contendo três pilares do desenvolvimento sustentável: o econômico, o ambiental e o social com ações em âmbitos globais, nacionais e locais ^36^.

A Tabela 1 apresenta dados que correspondem aos indicadores relacionados ao perfil demográfico, social, econômico, de recursos em saúde e características do sistema nacional de saúde.

**Tabela 1:** Comparação de indicadores demográficos, socioeconômicos e de saúde. Brasil, Espanha, Itália e Portugal, 2023.

Indicadores	Brasil	Espanha	Portugal	Itália
Demografia				
População (habitantes)	215.313.498	47.415.750	10.325.147	59.109.668
Extensão territorial (km^2^)	8.358.140	505.990	92.391	301.340
Crescimento demográfico (%)	0,50	0,10	0,30	-0,40
Economia				
PIB total (USD)	1,61 trilhão	1,43 trilhão	253,66 bilhões	2,11 trilhões
PIB *per capta* (USD)	7.507,02	30.103,50	24.567,50	35.657,50
% do PIB para despesas com saúde (p.p.)	10,30	10,70	11,2	9,60
Taxa de desemprego (%)	9,50	13,00	5,80	8,10
Social				
Coeficiente de Gini	0,44	0,34	0,34	0,35
IDH	0,76	0,90	0,86	0,89
Taxa de alfabetização (> 15 anos) (%)	94,30	98,60	96,80	99,30
Índice de incidência de pobreza (%)	5,80	0,90	0,50	0,80
Saneamento básico com segurança (%)	49,00	97,70	85,00	96,00
Prevalência de subnutrição (%)	4,10	2,00	2,50	2,50
Condições de saúde				
Expectativa de vida ao nascer (anos)	73	83	81	82,8
Taxa de mortalidade infantil/1.000 nascidos vivos	14,40	2,62	2,40	2,60

IDH: Índice de Desenvolvimento Humano; PIB: produto interno bruto; p.p.: pontos percentuais.

Fonte: Instituto Nacional de Estatística ^39^; Fundo das Nações Unidas para a Infância ^40^; Instituto Nacional de Estatística ^45^; Banco Mundial ^48^; Departamento de Informática do SUS ^49^; Programa das Nações Unidas para o Desenvolvimento ^50^; Instituto Nacional de Estatística ^51^; Organização Mundial da Saúde ^52^; Organização para a Cooperação e Desenvolvimento Econômico ^53^; Instituto Brasileiro de Geografia e Estatística ^54^.

Ao compararmos essas variáveis, há significativa diferença demográfica e de extensão territorial entre os países, evidenciando maior complexidade político-administrativa, intensa transformação estrutural e uma exigência máxima nas competências que cabem às instâncias de poder público e de gestão técnica governamental e política, no desatar dos nós que são consequentes desafios da disparidade loco regional, diante das diferentes potencialidades e dificuldades de cada estado/província. Nessa perspectiva, a qualidade dos sistemas de saúde é pautada mediante força de trabalho, no seguimento e no fluxo das redes assistenciais, bem como na prestação de serviços e na capacidade tecnológica e de conhecimento ^8^.

O desempenho do Estado na garantia de base estrutural adequada, no comprometimento e na vontade político-administrativa em traçar um projeto de nação com agenda forte para as necessidades da sociedade e que favoreça o ciclo produtivo do trabalho é fundamental para a capacidade de resposta de um sistema de saúde, quando considerado o conceito ampliado de saúde. Essas condições básicas de vida definem a capacidade das pessoas de enfrentarem seus problemas, o perfil de demandas nos centros de saúde e a consequente qualidade da prestação dos serviços ^2^ ^,^ ^4^ ^,^ ^37^ ^,^ ^38^.

Ao comparar os países, verificamos uma disparidade em relação ao coeficiente de Gini, ao Índice de Desenvolvimento Humano (IDH) e à incidência de pobreza. Essas dessemelhanças exercem influência expressiva sobre a eficácia dos serviços, pois são fatores decisivos para a configuração das demandas e o acesso à saúde ^2^ ^,^ ^7^. O Brasil enfrenta desafios adicionais devido à expressiva desigualdade econômica e ao tamanho territorial e populacional com maior incidência de pobreza, o que demanda abordagens estratégicas singulares de aprimoramento da equidade e da qualidade dos cuidados em saúde, sobretudo primários ^2^ ^,^ ^3^.

Países com coeficiente Gini mais próximo de zero, IDH mais elevado e, consequentemente, menor incidência de pobreza têm a vantagem de desfrutar de um maior acúmulo de conhecimento, anos adicionais de vida saudável e maior acesso a tecnologias em saúde que exercem um impacto significativo em sua longevidade e, consequentemente, proporcionam o acesso a serviços e tecnologias que aprimoram substancialmente a qualidade de vida da população. A fim de modificar o curso das desigualdades no desenvolvimento humano, torna-se insuficiente melhorar exclusivamente um ou dois indicadores específicos. É necessário realizar alterações nas estruturas sociais que perpetuam a iniquidade e, assim, promover a igualdade de oportunidades para que todos os indivíduos possam usufruir de um desenvolvimento humano pleno em busca de equidade ^35^ ^,^ ^36^.

Quanto ao crescimento populacional, os países europeus apresentam valores muito baixos, sendo que Itália, com negativo de 0,40%, e Espanha, com 0,10%, têm sido pontos de preocupação para o campo dos recursos humanos, suas competências e habilidades no que tange à qualificação e ao acompanhamento da inovação em saúde e da realização de tarefas que exigem novas performances no território e suas interfaces ^39^ ^,^ ^40^.

Outra variável de destaque é a abrangência do saneamento básico com segurança e qualidade. A garantia de saneamento básico adequado tem associação direta com a promoção da saúde e a prevenção de doenças infecciosas, com o fluxo dos serviços para atender as demandas agudas, sendo urgente o acesso de qualidade a serviços de: abastecimento de água; coleta e tratamento de esgotos; limpeza urbana, coleta e destinação do lixo; e drenagem e manejo da água das chuvas; o que favorece o avanço dos indicadores e exerce impactos positivos nas condições de vida e saúde das pessoas ^2^ ^,^ ^7^ ^,^ ^38^.

Sobre as condições de saúde, o comparativo é contrastante entre os indicadores selecionados. Para mortalidade infantil, as taxas são de 14,4 para o Brasil e 2,60, 2,62 e 2,40 para a Itália, a Espanha e Portugal, respectivamente. Em relação à mortalidade materna, a taxa é de 3/100 mil nascidos vivos na Espanha, 5 na Itália, 12 em Portugal e 72 no Brasil. No caso brasileiro, os valores ainda são muito preocupantes, apesar da redução nos últimos anos desses indicadores com a adesão do Sistema Único de Saúde (SUS) ao modelo assistencial saúde da família em 1994 ^40^. Esses achados apontam distintas conformações tanto na condição de vida quanto no estilo de vida dessas sociedades, deveres históricos do Estado para com suas populações, a depender diretamente do tipo de projeto político ideológico e de competência governamental na tomada de decisão da utilização dos recursos públicos a favor do desenvolvimento e da estruturação de uma nação, com compromisso de garantir subsídios básicos à dignidade humana, para que o sistema de saúde seja capaz de atender suas demandas.

Nesse caso, os países que começaram a reformar seus SNS a partir dos cuidados primários por volta das décadas de 1970 e 1980, como Reino Unido, Espanha e Itália, fizeram maiores progressos ^41^. No caso da mortalidade materno-infantil, uma APS concentrada na promoção da saúde com identificação precoce de doenças mostra-se mais preditora no controle da mortalidade prematura, sendo particularmente sensível ao diagnóstico precoce, cuidados longitudinais, ao território e à capacidade de coordenação entre os diferentes níveis da rede de atenção à saúde ^8^ ^,^ ^9^ ^,^ ^42^ ^,^ ^43^.

Em relação ao produto interno bruto (PIB) em saúde, os países tiveram um aumento no período pós-pandemia em 2022, com valores referentes de 11,2% e 10,7% para Portugal e Espanha e Brasil alcançando 10,2%, bem próximo à média da União Europeia que é de 10,8%. Já a Itália apresentou uma porcentagem inferior ao recomendado pela União Europeia: 9,6%. Investimento mais baixo e ascendência no envelhecimento da população geram desafios maiores às condições de saúde na Itália, afetada sobretudo pelo número insatisfatório de recém-nascidos ^44^ ^,^ ^45^ ^,^ ^46^. A reforma sanitária, iniciada em 2022 com o *Decreto nº 77* do Ministério da Saúde da Itália ^46^, propõe um novo arranjo assistencial com ênfase na organização e na aproximação do território com a expansão do modelo de saúde da família.

O investimento em saúde é essencial garantir maior bem-estar e qualidade de vida para as diferentes coletividades, devendo ser, portanto, uma política de Estado prioritária, em vez de meramente um ônus econômico para os órgãos governamentais. A efetiva alocação de recursos em saúde desempenha um papel crucial na redução da mortalidade e no aumento da expectativa de vida da população ^37^ ^,^ ^47^.

## Considerações finais

Este estudo aponta a necessidade de investimento para a melhoria dos indicadores socioeconômicos referentes à base estrutural, à proteção social e que determinam o índice de desigualdade, de desenvolvimento humano e de qualidade de saúde, o que não favorece o custeio e dificulta o alcance da eficiência das ações, dos serviços, da prática em saúde, no que tange aos sistemas de saúde de base universal e de atenção integral.

É imperativa a abordagem sobre a necessidade de uma base social estruturada e digna que consolide as condições de vida de uma população e sua relação direta com os DSS e o quanto ela é necessária no despertar de uma compreensão seguida de ação mais profunda e comprometida com os desafios que estão postos ao avanço das nações e à adoção de novas tecnologias promotoras de saúde para a APS.

As limitações se apresentam no imenso escopo de legislações de que trata um sistema público de saúde, deixando abertura para outras investigações que elucidem caminhos sustentáveis para a melhoria dos SNS com modelos ancorados na APS e na saúde da família a partir de outros olhares; buscando, assim, entender o cenário a que as sociedades estão expostas e apontar possibilidades para sustentabilidade de uma saúde universal, cada vez mais inclusiva, integral e equânime.

A inovação em saúde continuará acelerada; porém, o maior investimento tecnológico não está nos recursos materiais, mas sim nos imateriais de natureza e habilidades humanas que envolvam o processo de trabalho. Os países terão que se dedicar aos seus cidadãos com um grande senso de urgência, especialmente nas áreas de saúde e educação, que são os pilares do capital humano, para valer-se dos benefícios e atenuar suas piores rupturas ^47^.

O investimento contínuo na versão mais aparente dos problemas de saúde não alcançará grandes impactos e seguirá com avanços insuficientes e transitórios, com pouco favorecimento ao custo de efetividade do SNS e com uma capacidade transformadora frágil da qualidade de saúde e de vida das populações.
